# Cyber-Physical Systems and Smart Cities in India: Opportunities, Issues, and Challenges

**DOI:** 10.3390/s21227714

**Published:** 2021-11-19

**Authors:** Md. Onais Ahmad, Mohd Abdul Ahad, M. Afshar Alam, Farheen Siddiqui, Gabriella Casalino

**Affiliations:** 1 Department of Computer Science and Engineering, Jamia Hamdard, New Delhi 110062, India; oahmad@jamiahamdard.ac.in (M.O.A.); aalam@jamiahamdard.ac.in (M.A.A.); fsiddiqui@jamiahamdard.ac.in (F.S.); 2 Department of Computer Science, University of Bari Aldo Moro, 70125 Bari, Italy; gabriella.casalino@uniba.it

**Keywords:** CPS, smart cities, smart governance, smart economy, security

## Abstract

A large section of the population around the globe is migrating towards urban settlements. Nations are working toward smart city projects to provide a better wellbeing for the inhabitants. Cyber-physical systems are at the core of the smart city setups. They are used in almost every system component within a smart city ecosystem. This paper attempts to discuss the key components and issues involved in transforming conventional cities into smart cities with a special focus on cyber-physical systems in the Indian context. The paper primarily focuses on the infrastructural facilities and technical knowhow to smartly convert classical cities that were built haphazardly due to overpopulation and ill planning into smart cities. It further discusses cyber-physical systems as a core component of smart city setups, highlighting the related security issues. The opportunities for businesses, governments, inhabitants, and other stakeholders in a smart city ecosystem in the Indian context are also discussed. Finally, it highlights the issues and challenges concerning technical, financial, and other social and infrastructural bottlenecks in the way of realizing smart city concepts along with future research directions.

## 1. Introduction

There is no universal definition of a smart city [1]. Different regions connote its meaning differently. Every country has its own conceptualization of a smart city. It varies from country to country, city to city, and place to place [2]. Every country has their own set of requirements and abilities and thus has a different viewpoint and willingness to reform, which affects the migration from classical city setups to urban smart city settlements [3]. In this paper, we aim to give an overview of the smart city setups along with the opportunities, issues, and challenges associated with their successful implementation. With the rapid migration of inhabitants towards urban settlements, governments and policymakers face huge challenges [4]. A typical smart city setup involves facilities equipped with smart and state-of-the-art technology which includes smart transportation, smart energy, smart education, smart healthcare, smart sanitation and waste management, and smart governance, etc. [5]. In the present era of technological advancements, almost everything is converging into digitalization. In the same manner, the word “smart” is being applied everywhere and in every field [6]. With the rapid growth in the economy, the role of digitalization is highly critical to upgrade infrastructural support including physical, societal and institutional. This transformation is seen as a probable solution to several inherent and obvious city problems since it focuses on smart governance, smart IT and Communication, smart environment, smart buildings, smart businesses, and smart transportation, etc. [7]. Information Communication Technology (ICT) is considered the backbone of any smart city initiative; in the absence of ICT, imagining a smart city is baseless and will fail [8]. Smart city core infrastructural support systems such as sensor networks, IoT and wireless devices, and well-equipped data centers, etc., constitute vital infrastructural mechanisms that provide the key enabling services to the smart city. Smart cities are certainly the future habitat of citizens around the globe; hence, it will be a great infrastructural asset for them. Smart city projects can be thought of as an area of opportunity for all stakeholders, especially for the infrastructural companies as well as developers [9]. In large countries such as India, demographics and diversity pose great challenges as well as opportunities. Naturally, smart city projects will attract high investment and a network of state-of-the-art technology that will create environmentally sustainable solutions, smooth operational efficiencies, and better civic amenities for urban populations and citizens.

The rest of the paper is organized as follows. Section 2 discusses the concept of cyber-physical systems in the context of smart cities and the underlying security threats. Section 3 highlights the enabling technologies for smart city setups. Section 4 provides the opportunities, issues, and challenges associated with the successful realization of smart city ecosystems. The final Section 5 provides the discussion and conclusion.

### 1.1. Key Action Areas of Smart City

**Figure 1:**
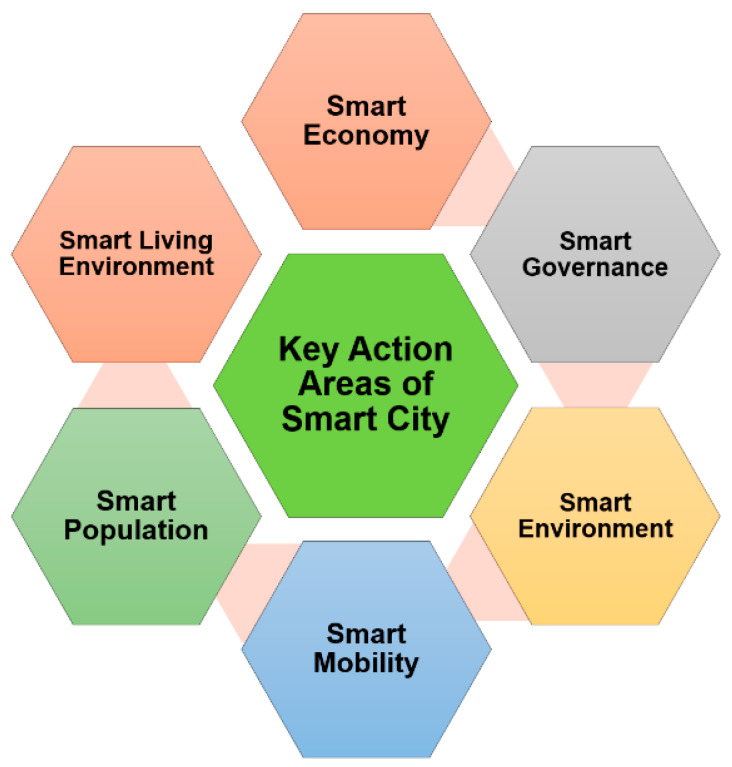
Key Action Areas of a Smart City.

A typical smart city consists of several key focus areas which need to be analyzed to provide holistic planning and designing. Figure 1 shows the key action areas of a typical smart city.

#### 1.1.1. Smart Economy

A smart economy relates to stimulating innovation as well as technological creativity based on scientific research, which cares for a smart environment and superior technology with an aim for attaining economic superiority and prosperity [10]. It further provides independence to a nation and creates equal economic opportunities for all stakeholders including inhabitants, governments, service providers, and other businesses, etc. [11,12,13,14].

#### 1.1.2. Smart Governance

Smart governance constitutes performing governance through digital means, a city where public services use digital platforms, e.g., wi-fi and other ICT-based online services. All the policies and projects are implemented, tracked, and maintained using digital technologies to maintain transparency, tracking, and security. Smart governance also provides flexibility in terms of dynamic updating of policies and regulations based on realtime data from the citizens in the form of feedback, suggestions, polls, etc. Smart governance follows the “citizen first” model of working where data-driven decisions are made to improve governance and involve greater participation by the citizens [15,16,17,18].

#### 1.1.3. Smart Environment

A smart environment is an environment with miniature sensors embedded in it, which continuously collect data on the subjects and their surroundings for providing bettering realtime insights into different atmospheric and other environmental parameters such as temperature, pressure, humidity, and other impurities present in the air, water, and soil. A smart environment is an essential part of any smart city. Such an environment promotes the use of ICT for taking automated self-corrective measures when the threshold limit of any parameter is exceeded [19,20,21,22,23]. The monitoring of the environment can be achieved through specialized CPS made for the purpose [24,25]. The specifically designed CPS makes use of the data collected through the IoT devices to perform analytics and make data-driven decisions according to the current needs [26].

#### 1.1.4. Smart Mobility

Smart mobility aims to automate classical transportation management through the use of sensors embedded in traffic signals, street lights, zebra crossings, etc. The concept of smart mobility ensures smooth traffic management and dynamic handling of traffic congestions, providing safe, secured, and free passage to emergency services, including ambulances, police vehicles, government vehicles, etc. Optimal routes are identified and conveyed for effective management and mobility of the masses. Smart mobility ensures pollution-free, environmentally-friendly, and congestion-free mobility leading to an effective means of sustainability in travel and transportation. CPS can be effectively used in smart mobility solutions to provide fault-tolerant and robust transportation across the smart city [27,28,29].

#### 1.1.5. Smart Population

A smart population includes citizens participating in the development of the nation including various initiatives of the government in an environmentally-friendly, secure, and privacy-preserved manner. There should not be any bias, digital divide, or other forms of inequality concerning age, race, caste, or creed. CPS can be used to build intelligent and smart communities. The data collected and shared between different stakeholders through the CPS can be used to provide deeper insights into the real challenges and issues faced by the inhabitants [30,31,32].

#### 1.1.6. Smart Living Environment

The concept regarding a smart living environment is related to good health and a hygienic environment. CPS can aid in the development of automated smart living spaces where the errors of the system or networks are mitigated automatically based on self-learning through AI/ML algorithms. These smart spaces are self-reliant and can scale as and when required to accommodate more and more entities. Since every aspect is largely automated, it is easier to identify and diagnose a fault, track the progress, as well as trace the system logs to identify any irregularities [33,34,35].

Apart from these, there are other areas that are of utmost importance to smart cities. These include smart energy and smart manufacturing. Smart energy systems aim to provide a clean source of energy to the users with minimum or no negative impact on the environment [36,37,38]. These include energy from renewable sources such as solar, etc. Smart manufacturing refers to adopting ICT for automation of all aspects in the manufacturing domain [39,40].

### 1.2. Major Contributions

The following are the major contributions of this paper.

- Comprehensive analysis of the CPS, highlighting its working mechanism, application areas, opportunities, issues, and challenges in its realization.
- Highlighting the advancements in CPS from an Indian perspective.
- Identifying the role of various enabling technologies in a smart city with regard to cyber-physical systems.

### 1.3. Smart Cities-Indian Perspective

**Figure 2:**
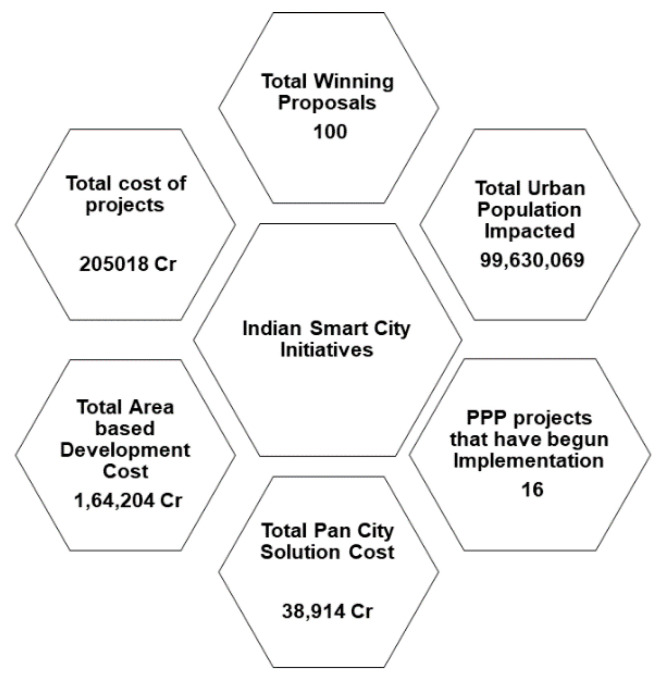
India’s Commitment towards Smart City Initiatives.

The concept of smart cities in India poses several different kinds of issues and challenges. The size of the nation and the diverse population make India a unique case for smart city setups. Most of the existing cities are unplanned and have several issues of waterlogging, sanitation, proper water supply, etc. Tackling all these issues is a very challenging task. In a typical smart city architecture, cities are divided into different areas for better governance, administrative, and utility purposes, and each one keeps its service outlets [41]. The Indian government initiated the mission of smart city development in 2015 (https://smartcities.gov.in/. Accessed on: 12 September 2021). Initially, 20 cities were selected for the mission through an open competition. The core aim of the mission was to provide an underlying infrastructure and quality of life to the inhabitants of the city through sustainable, cost-effective, efficient, and environment-friendly measures. The development was planned by identifying key areas within the city limits and, in parallel, focusing on each selected area of development (http://mohua.gov.in/#skip. Accessed on: 14 September 2021). Figure 2 gives an overview of India’s commitment to smart city initiatives (https://smartnet.niua.org/mis/drupal/scm.php. Accessed on: 17 September 2021).

## 2. Cyber-Physical Systems (CPS) in Smart Cities

**Figure 3:**
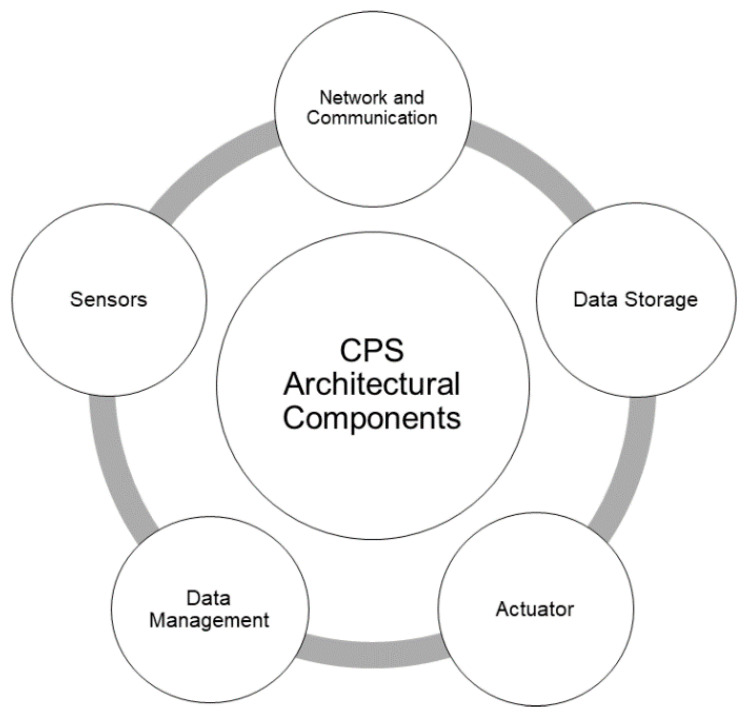
Architectural components of Cyber Physical Systems (CPS).

Cyber-physical systems (CPS) were introduced in the year 2006 by Ellen Gill. The concept was based on the combination of cyber (computation and communication) and physical components (devices, gadgets, and systems) that can use smart computation techniques to interact with real-world objects [42]. Figure 3 shows the architectural components of a CPS [43,44,45,46].

- Actuator

The role of an actuator in a typical CPS is to convert the control command into mechanical work. The actuators may be susceptible to hacking where the illegitimate hackers can take control of the actuator device to alter the mechanical work being carried out. This may include locking/unlocking doors, starting, stopping, or changing the speed of clinical wearables, etc. Actuators are the actual controlling mechanism behind the CPS. Since there are multiple resources and services to be controlled, the choice of selecting an optimal actuator plays an important role. There are several actuator-selection approaches as discussed in [47,48].

- Data Management

This module is responsible for managing the data that are being sensed by the sensors embedded in the different IoT devices in the CPS. This may include data cleaning, preprocessing, data standardization, normalization, etc. Effective management of data generated from sensors is highly crucial for extracting value and taking optimal decisions based on that data. Several data management issues that need to be resolved include selection of appropriate data management systems, data cleaning, and removing data outliers (if any), and security of data at rest and in transit. Apart from these issues, the choice of data management systems also depends upon the type of activity and application [49,50].

- Sensors and Sensing Module

This module is responsible for sensing the data about the subject and its surroundings. The sensed data are transferred to the data management unit through the internet gateways. Wireless sensors networks (WSN) are the backbone of any CPS. The collection of multiple sensors in the WSN works in synchronization to integrate and perform the task of sensing the data. The data thus collected contain much deeper insights that were not possible with individual sensing capabilities [51,52]. Furthermore, optimal dynamic network topologies can be adopted to place the sensors at appropriate positions within the CPS ecosystem to cater to the needs of changing subjects and their surroundings. The concept of relay nodes is also adopted to provide multihop communication reducing the energy required for data transmission [53].

- Network and Communication

This module consists of communication protocols for establishing the connection among different entities within the CPS. This module governs all the communication including routing, rerouting, error handling, acknowledgments, etc. Several state-of-the-art approaches can be adopted for managing the communications within the CPS ecosystem. With a wide variety of diverse devices connected in a typical CPS, there are several issues and challenges, which include device schemas, data capturing formats, communication protocols, energy efficiency, etc. [54,55,56]. To overcome these issues several robust approaches have been proposed in the literature. In addition, SDN and NFV technology can also be adopted to control network communication more flexibly and optimally. SDN and NFV approaches provide a software-based controlling of the network parameters including congestion control, route management, placement of relay nodes, number and types of network hops, etc. [57,58].

- Data Storage Module

This module contains cloud and edge-based storage services where the sensed and cleansed data finally reside. It also includes cached data and metadata records. A typical CPS generates huge volumes of rapidly expanding big data. This massive amount of data needs specialized tools and techniques for storage and management. Several big data storage and management approaches, for example, Hadoop, Casandra, HDInsight, NoSQL, etc., can be used. It is generally considered appropriate to partition the data into smaller portions and store it in a distributed manner with an optimal number of copies to provide better control and immediate availability as and when required [59,60]. In [61], the authors proposed a large-scale framework for realtime monitoring in CPS. They used industrial CPS as a use-case to evaluate the effectiveness of the proposed approach.

### 2.1. Working of CPS

**Figure 4:**
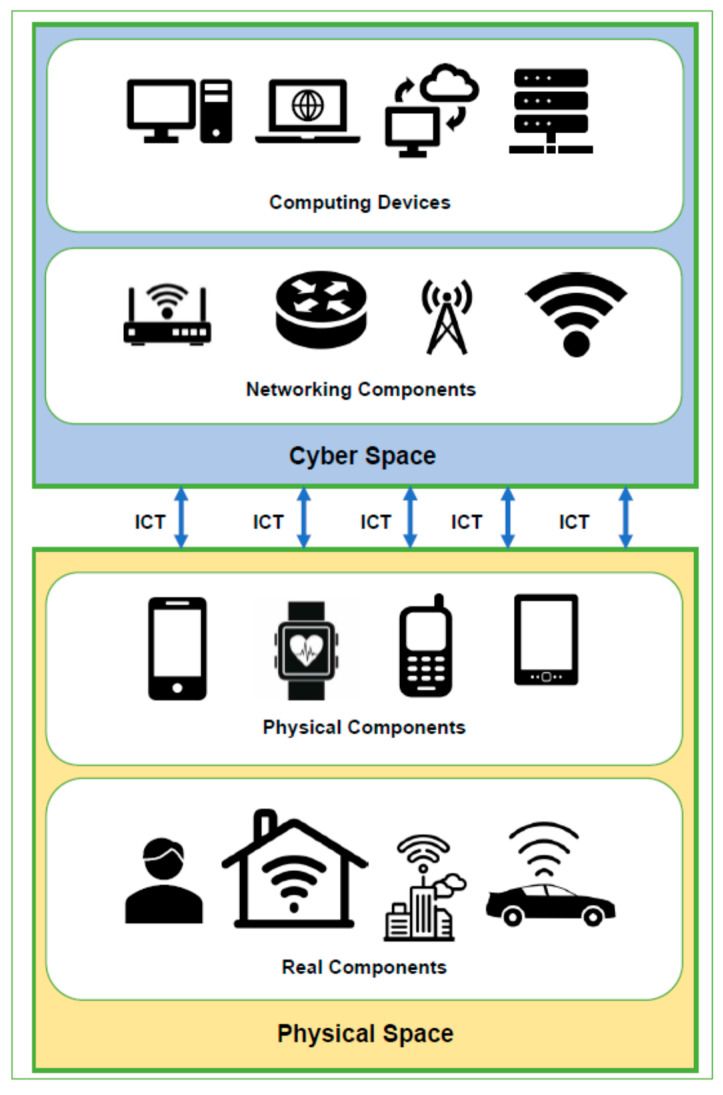
A typical CPS Diagram [45].

A typical CPS system tends to integrate physical components with the computational components present in cyberspace through a network. Once a successful connection is established, the physical processes can be controlled and managed through software programs. CPS provides abstraction by modeling the dynamics of physical processes with the help of software. This automated controlling of physical processes is improved by providing feedbacks through data generated by different connected entities within the CPS. As shown in Figure 4, a typical CPS can be considered as an embedded system with computations, communications, and control capabilities. In a transportation management CPS, the realtime data of the traffic conditions are captured through sensors embedded in the roads, traffic signals, footpaths, sign boards, etc. The number of vehicles passing through the footpaths or at a stationary position can be measured through the pressure measuring sensor chips embedded in the footpaths. Similarly, inductive loop detectors sensors are used to detect the number of vehicles arriving at any point on the road. The acoustic and ultrasonic sensors are used to measure pressure and sound waves produced through the friction between vehicle tires and roads lanes for identifying the speed and lane detection. These data are then fed into the CPS system to take dynamic decisions for updating traffic signals, enforcing speed limits, giving free passage to emergency services vehicles (if any). In addition, the duration of the signals is also managed dynamically based on the traffic conditions to manage the smooth flow of vehicles without congestion. Apart from the sensors embedded on the roadside signals, footpaths, and signboards, the users (as an entity of the CPS) also share data about realtime traffic conditions and alternative routes with the system to provide optimal management of traffic. Möller and Vakilzadian [28] discussed the different components of CPS for smart transportation. They showcased the importance of vehicle-to-vehicle communication, realtime feedback for traffic conditions, and the integration of information from different entities. Kukkala et al. [62] provided a survey of advanced driver assistance systems (ADAS). They described different hardware-based sensors and corresponding software used in ADAS highlighting the pros and cons of each. In similar ways, the environmental cyber-physical systems (ECPS) are designed to monitor the ecological conditions of the subject and its surroundings. The subject can be any indoor unit (house, factory, building, etc.) and the surroundings include all the nearby outdoor areas of the subject. Criado et al. in [63] proposed a web socket-based approach for the integration of various heterogeneous components in a smart home environment to ensure interoperability and seamless connectivity. Through the help of smart dust (minute sensors based microelectromechanical systems) the different parameters of the environment such as light, temperature, pressure, humidity, composition of gases, magnetism, and chemicals, etc., can be sensed and detected [64]. Smart dust works on the principle of IEEE 802.11 b/g standards for wireless communications [65]. Different kinds of sensors such as trace metal sensors, radio isotopic sensors, compound detection sensors, etc., are used to detect the presence of environmental pollutants in the air, water, or soil [23,66]. The data collected from such sensors are processed and analyzed to take appropriate countermeasures. Similarly, motion detection sensors and temperature sensors are used in indoor units to control the different devices and appliances based on user preference and conveniences. This may include automatic controlling of AC units, turning off/on the lights, fans, and other appliances, with respect to user availability and preferences.

**Figure 5:**
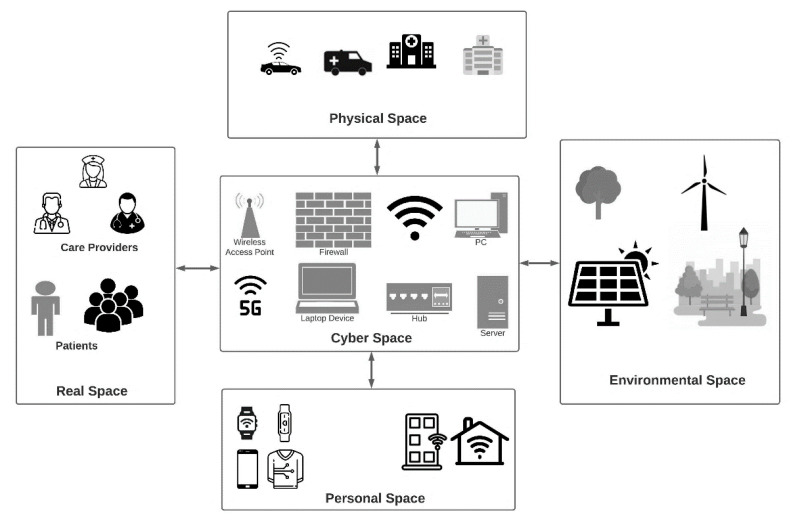
A typical medical CPS [45].

To explain the working of cyber physical systems, we use the example of the healthcare domain. The Medical CPS (MCPS) can collect and process data from the clinical and wearable sensors worn by the patients. The biosensors have the potential to sense the critical physiological parameters of the patient and send them to the computing and analytics unit for further processing. There are multiple advantages of biosensors, which include noninvasive delivery of drugs (in the form of smart pills) and sensing of blood glucose parameters. Hussain and Park [67] proposed a portable EMG-based gait monitoring system. They further identified the effectiveness of myoelectric biomarkers for the classification of stroke-impaired muscular activity. Similarly, there are several other wearable devices such as EMG, ECG, and EEG devices, and sensors such as blood flow sensors and chemical sensors for identifying chemical concentration, PH value, and glucose concentration in the blood. Force sensors are used in kidney dialysis devices. The biosensors are used to sense enzymes, antibodies, and other microbes within the human body. Petropoulos et al. in [68] discussed an IMU-enabled posture monitor for identifying the wrong sitting posture through motion sensors attached to the back of the users. These devices and sensors are used in the medical CPS to provide valuable information about the condition of the patients. With medical CPS, the sensed data are cleaned, standardized, and finally forwarded to the processing and analytics unit to perform analysis. The type of processing depends upon the services required. For example, for realtime requests and queries, the data need to be processed as close to the source (of data generation) as possible. It is vital to provide an immediate response for the requested services. Further, processing the data close to the source reduces network latency and communication bottlenecks, if any. To do so, the MCPS uses an edge computing paradigm in which the sensed data are processed at the edge of the network to provide realtime or near realtime services [69]. Similarly, for non-immediate requests and services, the processing is completed on the cloud. Since moving the sensed data to the cloud is a bandwidth-hungry process, optimal routing and congestion controlling mechanisms are needed to reduce the time of data transfer. Wang et al. in [70] proposed an edge computing-based approach for mitigating coupling issues in CPS. The different wearable point-of-care devices, which are also equipped with miniature sensors are used to provide monitoring and analysis on the go or at homes as well [71,72,73,74,75]. Figure 5 shows a typical medical CPS.

A medical CPS as a whole consists of several components which include sensing, analysis, security, storage, and management. Each of these units has its specific functionality. The data collected from the patients in some cases are highly sensitive and thus must be protected from any kind of theft or hacking. To do so, the medical CPS makes use of different cryptographic techniques to encrypt the data before sending them across the network. Casalino et al. in [76], proposed the concept of fuzzy inference systems in telehealth for critical disease care. Similarly, there are several data management and storage techniques employed within the CPS, which provide optimal data storage and fetching as and when required by the care providers.

With the advancement in technologies including IoT, WSN, big data, and enhancement of computational capabilities, the concept of CPS is widely implemented across multiple domains. The applications of CPS can be found in diverse areas such as aerospace, healthcare, energy, transportation, manufacturing, etc. [77,78,79,80,81]. Smart city ecosystems can be viewed as a large-scale CPS implementation that facilitates the cooperation between various computational, communication, and physical aspects and also helps to provide a better quality of life. These CPS are an integration of components of different natures, which aim to control, manage, and monitor a physical process and also adapt to the changes based on the feedback. A CPS can be thought of as a driver of the smart city services having the capability to completely transform the way of life of the inhabitants. A CPS may be used to collect and share data about realtime traffic conditions, health conditions of the patients, environmental phenomenon, land-use planning, air/water/soil quality, structural health of buildings, roads, and other structures such as bridges, rail tracks, monuments, etc. Since it involves a complex integration of multiple miniature devices (sensors, actuators, and ICs) and larger devices (mobile phones, servers, and clouds), securing such systems is a very challenging task. There are always chances of data leakage and or security breaches from one or the other vulnerable systems or devices. Therefore, it is of utmost importance to implement granular and layered security mechanisms for such types of complex CPS. MFA can greatly help in providing an extra layer of security to the CPS, but we need to make sure that implementing this extra layer of security is not an overhead and does not increase latency, thus compromising the primary aim of the CPS systems. Table 1 provides the applications of CPS in different domains.

### 2.2. Security Threats in CPS with Respect to Smart Cities

Smart cities incorporate a broad range of cyber-physical systems (CPSs) to boost their efficiency by minimizing expenses and resources. These CPSs, such as smart healthcare, smart transportation, and smart grids, interact with the residents in an active, coordinated, and reliable manner, thereby strengthening the public infrastructure and lowering the living costs [131]. The incorporation of CPSs in smart cities aims to enhance transport, infrastructure, healthcare, safety, and other utilities, but these enhancements result in an elevated risk and susceptibility [132]. Moreover, linking additional devices to the current CPSs introduces additional security threats. The rapid use of CPSs in critical frameworks such as medical equipment and defense puts human lives at serious risk, owing to cyberattacks. In the context of smart cities, some of the security threats prevalent in CPSs are given below [130,131,132,133]

- The deployment of ICT-based smart vehicles enables malicious individuals to gain control of the automobile, endangering the lives of the driver and other passengers [131] The attacker might ask for a ransom to release control of the automobile. Similarly, hackers may encrypt important files on a CPS-connected device and ask the owner for a ransom to grant access (ransomware).
- The lack of security updates in CPS linked devices, together with device misconfiguration and the prevalent use of default passwords and settings, as well as the absence of encrypted communication between devices, also presents a serious security loophole. Similarly, poor credentials threaten the security of both the user and their business, as cybercriminals can set up remote sessions to track them. These intruders can identify a user’s physical location by using IP addresses or GPS modules of the CPS devices [130,131,132].
- In a smart city, the prevalence of “actuators” that regulate the physical skeleton (filters, heating components, faucets, filters, etc.) renders the city vulnerable to physical damage if the systems are compromised [132,133].
- Artificial intelligence applications in CPSs are susceptible to data manipulation attacks where cybercriminals can steal sensitive information to generate superficially legitimate input to mislead the algorithm. These attacks could be driven by financial or political rivalry, powered by the tremendous expansion of built-in computing capacity.
- Hackers could seize control of CPS linked devices and use them to disrupt business activities, use them as spam email servers, or turn them into botnets for carrying out DDoS (Distributed Denial of Service) attacks. Individuals with malice can access all smart appliances such as televisions, cameras, and refrigerators, and transform them into attack carriers.

Emerging technologies such as software-determined networks (SDN), blockchain, and game theory have surfaced as promising solutions to alleviate the threats described above. Additionally, it is crucial that the vulnerabilities stemming from deployment settings and implemented technologies be addressed at the initial stages of design and individual security roles be defined for cybersecurity staff and administration in a smart city. Table 2 provides the summary of security attacks and their corresponding mitigation measures adopted in recent literature.

### 2.3. Current Developments in CPS in India

With the massive population and area of coverage, the Indian subcontinent poses its unique challenges. The administration in India is keenly focused on realizing the true potential of CPS. Many flagship programs are being organized which have invited academia and research institutions across the country to work on the development of interdisciplinary CPS (https://serbonline.in/ICPS/HomePage. Accessed on 15 September 2021). The national mission on ICPS is focused primarily on the development of cyber-physical systems to solve country-specific problems through the development of embedded systems using IoT for smart homes and services, promote entrepreneurship, social inclusion, etc. (https://nmicps.gov.in/Home/ICPSNMHOME/HomeNM. Accessed on 18 September 2021) One of the premier institutions in India IIT Guwahati launched Intelligent CPS for developing indigenous technologies (https://timesofindia.indiatimes.com/gadgets-news/iit-guwahati-launches-centre-for-intelligent-cyber-physical-systems/articleshow/86295156.cms. Accessed on 17 September 2021). Apart from these, there are several other initiatives started in India for developing state-of-the-art solutions for tackling societal issues and problems utilizing the concept of CPS.

## 3. Role of Technology in Smart Cities

In a smart city, technological literacy or knowledge is a significant factor for bringing intelligence to the city entities and services. This concept not only provides smart services to its citizens but also plays a pivotal role in good governance, as it provides inputs/feedbacks to the government [144]. Thus, it can be concluded that all these things cannot be achieved without technical support and facilities [145]. Another important technology that is highly significant for a smart city setup is two-way communication. It enables the interaction among different entities of the smart city ecosystem. Keeping the city’s requirements, the government can plan and build that city in line with the citizen’s needs. ICT, acting as synergic resources for a dynamic communication system between the citizens and the government enables government agencies to have very clear analytical views of the demand pattern of the citizens. ICT also helps in the collective intelligence (CI) that can be established for the optimization of resources with the support of analytics and deep learning [146].

### 3.1. Internet of Things

Internet of things (IoT) may be termed as the veins of the smart city as it is present in all entities of the smart city ecosystem and connects each dot (node), as every device of the smart city is required to be interconnected [146,147,148]. Such arrangements make it possible for the devices to connect and communicate amongst themselves and make decisions for the efficient management of the smart city services. Almost all smart cities are based on IoT for deciding and acting.

### 3.2. ICT and Sensors

It is imperative to have proper knowhow of the underlying technology for smart city development. Modern-day technologies such as IoT, deep and machine learning, geospatial, ICT, sensors, etc., are all needed for a comprehensive smart city experience [149]. Undoubtedly, ICT technology is a thread that can channel all data from every entity and stakeholders including utilities, waste management, better healthcare, etc., to surveil or monitor to provide improved services [150,151,152].

### 3.3. Artificial Intelligence

Artificial intelligence is considered the most important technology for smart city setups. With the ability to perform automated, data-dependent, precise, and human-independent decisions, AI systems are widely used in smart city ecosystems [153]. The various services of security surveillance, traffic management, healthcare, and governance, etc., are all being improved with the help of AI technology [154,155].

### 3.4. Blockchain Technology

Blockchain technology is applied in several domains including healthcare, supply chain, resource management, tracking and tracing, identification and authentication, etc. In the smart city ecosystem, blockchain technology can be used to provide improved authentication of entities, users, and services [156,157,158,159,160,161,162,163,164].

### 3.5. 5G and SDN Technology

The advancement in 5G technology has opened a new horizon of opportunities for the smart city ecosystem. Bandwidth-hungry tasks can be more easily carried out seamlessly through the use of 5G networks. 5G technology further provides better connectivity and faster data transmissions needed for realtime or near realtime applications and services [165,166,167]. The ability to control and manage the network dynamically through SDN provides flexibility in terms of improving the scalability and dynamic routing and rerouting of data packets in case of any hindrances in the network [168,169,170].

### 3.6. Deep Learning

Deep learning approaches provide deeper insights into the system and its components making it easier to take better and informed data-driven decisions. The unparalleled data analyzing capabilities of deep learning technology aid in the pre-identification of any errors or future conditions of the systems and their components to provide predictive analysis and maintenance of the faulty components [171,172,173].

## 4. Opportunities, Issues and Challenges in Smart Cities

This section provides the various opportunities, issues, and challenges associated with the realization of smart city initiatives [4,5,6,7,79,83,132,133,134,135,136,157,158,159,160,161,162,163,164,165,166,167,168].

### 4.1. Opportunities

**Figure 6:**
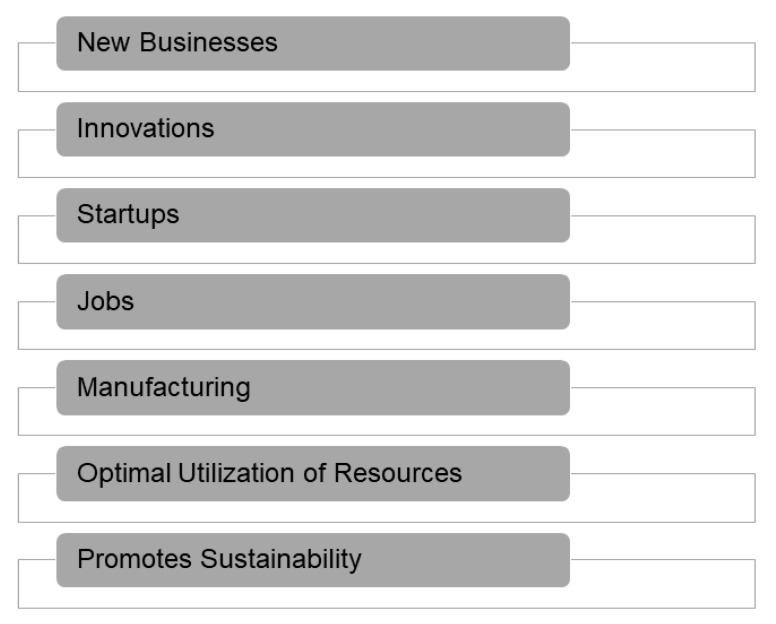
Opportunities of Smart City Initiatives.

Since India is a very large country in terms of area as well as population, there are several opportunities for stakeholders in smart city initiatives. Since there are large cities needing to be developed, there is consequently a large amount of work involved in the planning, development, and maintenance. The cost of such projects will be huge as already shown in Figure 5. Moreover, such projects will create job opportunities for several classes including skilled and unskilled laborers. Apart from this, many manufacturing and building construction opportunities will be created for medium and small-scale industries, apart from the MNCs. The development of smart cities serves two purposes. Firstly, it promotes reduction in resource usage, and secondly, it reduces resource wastage. Figure 6 highlights some of the opportunities of smart city initiatives [4,9,77,78,79,80,81,174].

#### 4.1.1. Businesses and Manufacturing

With the large-scale adoption of smart city projects, MSME businesses are bound to grow. Raw materials, spare parts, construction materials, operational and maintenance infrastructure, etc., will provide new business opportunities for all small or large market players alike [174,175].

#### 4.1.2. Jobs

The massive setups will require a large workforce at all levels for the completion of smart city projects. Skilled, semi-skilled, and unskilled labor will be required, and thus a large job market will be created with the large-scale adoption of smart city projects [176,177].

#### 4.1.3. Innovations

With sustainability at the core of smart city initiatives, legacy approaches will be replaced by new and innovative approaches and mechanisms for performing tasks. Thus, it will promote and boost innovative capabilities. The Government of India is already aware of this fact and therefore massive national level open challenges and competitions are arranged where they are inviting innovative solutions for the grassroots problems faced by the society, for example, Smart India Hackathon (https://www.aicte-india.org/Initiatives/smart-india-hackathon. Accessed on 29 October 2021), Atal Innovation Mission (https://aim.gov.in/. Accessed on 29 October 2021), Drug Discovery Hackathon (https://www.mygov.in/task/drug-discovery-hackathon-2020/. Accessed on 29 October 2021), etc.

#### 4.1.4. Startups

There are several issues and challenges that exist in classical city setups. For all such issues and challenges, the concept of startups has emerged in recent years in India. These startups identify the grassroots level basic problems of the society and provide innovative solutions to such problems. Smart city initiatives will surely boost the startup culture as there will be several intrinsic and extrinsic problems associated with such massive ecosystems, which may be hidden initially but may arise as the concept matures. Therefore, startups will be focused on solving such issues for providing better services to the inhabitants. The government is also focusing on promoting startup culture with several flagship initiatives, for example, Startup India (https://www.startupindia.gov.in/ Accessed on 30 October 2021), etc.

#### 4.1.5. Optimal Utilization of Resources

One of the most crucial opportunities that smart city initiatives bring is the optimal utilization of resources. With intelligent and smart data-driven measures, city resources can be optimally utilized by the inhabitants and it can further promote a reduction in the wastage of valuable resources, for example, smart water meters can reduce water wastage, the smart grid can reduce electricity consumption, etc. [178,179].

#### 4.1.6. Promotes Sustainability

The prime aim of smart city initiatives is to promote sustainability in every aspect. The data-driven mechanisms for different purposes through sustainable means are the key driving forces for attaining overall sustainability [180,181].

### 4.2. Issues and Challenges

**Figure 7:**
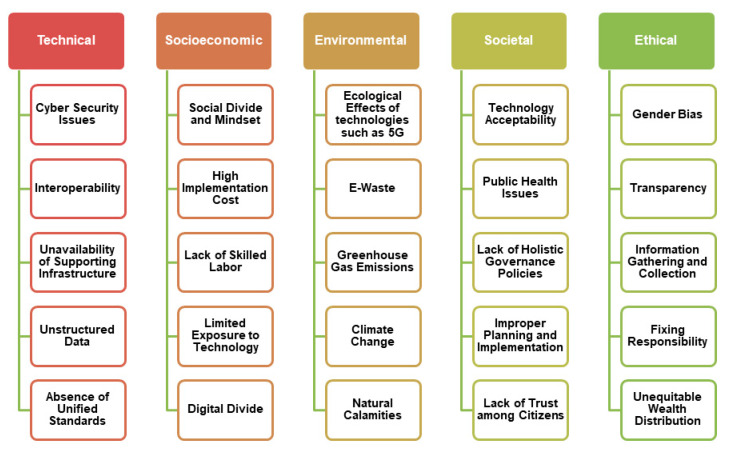
Issues and Challenges in Smart City Development.

There exist very unique issues and challenges in the development of smart city initiatives. They are broadly classified into five categories namely technical, socioeconomic, environmental, societal, and ethical, as detailed in Figure 7 [144,145,146,147,148,149,157,158,159,160,161,162,163,164].

#### 4.2.1. Technical Issues

These issues comprise a lack of infrastructure, scarcity of technical knowhow, privacy, and security issues [4,77,78,79,80,81,133,134,144,157,164,165,172,173,174,175,176,177,178,181,182,183,184,185,186,187,188].

##### Lack of Infrastructure

The smart city ecosystem is heavily dependent upon a state-of-the-art infrastructure consisting of sophisticated network devices, sensors, IoT devices, heavy-duty servers, security devices, etc. These devices are not readily available or they are too expensive for some nations who have to search for more economical alternatives. The architectural scalability must pertain to the smart city so that processing of data and also analytics requirements could be increased. The critical system cannot afford downtime as it needs high availability.

##### Scarcity of Technical Knowhow

There is an acute shortage of skilled labor to handle the complex underlying systems of the smart city ecosystem. The available workforce is not well-versed in the technical knowhow of the new devices and thus cannot effectively handle those devices.

##### Privacy and Security

Privacy and security are the two most important issues associated with smart city initiates. Since all the data are available in digital form and stored on the cloud (mostly), its privacy and security are always questionable when a third party is involved. Several approaches have been proposed in the recent literature to mitigate the security issues in CPS within smart cities [52,60,119,122,124,129,131,132,133,134,135,136,137,138,139,140,141,142,143].

##### Interoperability

The majority of IoT manufacturers focus on providing immediate services to their users rather than focusing on other internal details including security, interoperability, etc. These IoT devices are generally proprietary devices and lack interoperability, which limits their use [187,188].

##### Unstructured Data

The vast amount of data generated through ubiquitously available sensors is usually unstructured, which makes it very hard to handle effectively. Different data cleaning and structuring techniques are required to preprocess the data before feeding them to the decision-making unit. Since the services and functionalities of typical smart cities are data-dependent, the veracity and trustworthiness of the data are highly vital [189].

##### Absence of Unified Standards

To the best of our knowledge, there are no unified standards available for handling sensors and IoT devices deployed in CPS. The lack of unified standards makes it difficult to provide seamless integration and communication among various participating entities of the CPS systems in smart cities.

#### 4.2.2. Socioeconomic Issues

These issues comprise budgetary constraints and rigid policies in place to implement smart city initiatives.

##### Budget Constraints

Several smaller nations are not able to bear the costs involved in setting up smart city ecosystems and thus are not able to start the initiatives. There are several situations where nations have started initiatives but have to keep the project on hold due to lack of funds.

##### Rigid Policies

Legacy policies of nations are also considered a hindrance to smart city initiatives. The lack of a single-window clearance system, rigid governance, and regulatory norms are inherent issues that need to be tackled to provide a flexible and open system of clearance for such innovative projects.

#### 4.2.3. Social Issues

In a country like India, which is so diverse in terms of religion, color, caste, and creed, social issues are one of the most prominent factors influencing the decisions of the authorities and citizens alike. Dense population, conventional mindset, illiteracy, and digital divide are the prime factors that hinder smart city projects in this context.

##### Social Divide and Mindset

The conventional mindset and social divide also hinder the widespread adoption of smart city initiatives. Generally, older people are reluctant to adopt new technologies because of the fear of data theft or lack of technological knowhow.

##### High Implementation Costs

The implementation cost of smart city initiatives is usually very high. This is a serious issue that prevents smaller nations from readily adopting these initiatives. The CPS systems are highly costly and sophisticated and require expert handling and management.

##### Lack of Skilled Labor

There is a scarcity of skilled labor capable of handling the sophisticated CPS systems within the smart city ecosystems. Since every CPS is made up of thousands and millions of IoT devices and sensors, it becomes very difficult to manage them effectively without the help of trained professionals.

#### 4.2.4. Environmental Challenges

There are several hazardous effects of technology such as carbon emissions, e-waste, etc. These are termed as “environmental challenges”. Research is ongoing across the globe to study the negative effects of technologies such as 5G and 6G, in terms of E-waste generation, carbon emissions, etc. [190]. Other environmental challenges include natural calamities such as earthquakes, floods, lightning, snowfall, draughts, etc.

#### 4.2.5. Societal Issues

These issues pertain to common citizens, which include technological acceptance, public health, lack of knowledge about the existing policies, schemes, and regulations, and lack of trust among citizens.

#### 4.2.6. Ethical Issues

These issues are usually ignored by the majority of policymakers. However, in recent years, ethical issues have been an emerging topic of research. With the adoption of automation in several service domains issues of unconscious gender bias, transparency, rights of the machine, fixing responsibility in case of any mistakes, etc., are hard to identify and mitigate. Other ethical challenges include fear of job loss, unequal distribution of wealth among the stakeholders, dependency on machines, etc. From the smart city perspective, ethical issues play a vital role in governance [191,192]. Self-awareness and education about the pros and cons of technology must be considered while using smart city services.

## 5. Conclusions and Discussion

The concept of a smart city has gained ground in recent years across the globe. It is most likely that it will continue in the future. Modern-day smart technologies are providing solutions such as saving money, maintaining a sustainable environment by reducing carbon emission, reducing capital and operational costs, promoting better wellbeing and livelihoods, etc. Rapid and unprecedented urbanization severely posed traffic congestion. Consequently, air pollution became a big problem for cities. Transportation and traffic snarls led to another major issue in the transportation system. However, it is hoped that it may be eliminated by succinctly applying the principles of smart city concepts. Smart infrastructure responds intelligently to changes in its environment, including user demands and other infrastructure to achieve better and improved performance. The data-driven approach of smart cities provides exemplary benefits in all spheres of life for the inhabitants. “Data”, being the nerve center, means governments must collaborate and coordinate with start-ups and entrepreneurs to obtain effective collaborative outcomes which can be a win-win situation for all stakeholders. It is quite obvious that smart city networks are based on execution, not just planning. It is not an exaggeration that smart city projects can only be achieved when we intersect them with the basic infrastructure. The concept of a smart city has many benefits to its stakeholders if it is a data-driven, robust, dynamic, scalable, and responsive ecosystem. If we have an effective channel of communication, it would automatically connect each stakeholder for improved connectivity and synchronization. Cyber-physical systems being the building blocks of any smart city ecosystem, extensive research is being carried out around the globe to improve their performance, associated security, and privacy issues and improve the maintenance and lifetime of the systems. In recent years, India has also focused on realizing the adoption of CPS in different domains within the smart city framework for the improved management of services with optimal utilization of resources and sustainability as a prime focus. Extensive research and initiatives are being started to take up the challenge.

The enabling technologies facilitate an effective and cost-effective mechanism, thus, applying all the available technologies at our disposal, we can make robust networks of infrastructure and services making an excellent smart city ecosystem to provide better wellbeing and sustainable livelihoods for the inhabitants. Future research must be focused on the security and ethical aspects of these CPS for their widespread adoption in mainstream city developments. One of the latest technologies slowly coming into the mainstream is the passwordless authentication approach where the users are not required to memorize a username or password, and the authentication takes place with the help of some hardware keys, biometrics, or login tokens, etc. It will be interesting to see its application in the CPS domain as well.

## Figures and Tables

**Table 1:** Application areas of CPS.

S. No	Smart City Component	Application	Ref
1	Manufacturing	The manufacturing process can be automated to optimize the productivity of goods and enhance the delivery of services.	[82,83]
2	Healthcare	Smart Healthcare systems are designed for realtime monitoring of patients and support remote healthcare services using semiautomatic medical devices.	[76,84,85,86,87]
3	Transportation	A smart transportation system in a smart city uses embedded sensors for realtime information sharing and processing for traffic management. Advanced sensing, communication, control, and computations enable the efficient working of autonomous vehicles.	[88,89,90,91,92]
4	Energy	The integration of cyber and physical systems helps to provide a reliable, safe, and secure supply of energy and led to the development of smart grids.	[93,94]
5	Infrastructure	The use of sensors in buildings helps to minimize the overall cost of functioning by optimizing the processes based on the data analysis and feedback mechanism.	[95,96]
6	Agriculture	Regular monitoring of environmental conditions helps to improve agricultural production. Using IoT and sensor technologies helps to implement smart water management, soil monitoring, and efficient supply chains.	[97,98,99]
7	Education	CPS implementation into conventional education systems can help to develop smart learning environments where all entities can share information and data.	[100,101,102,103,104,105,106,107,108,109]
8	Business	The integration of smart techniques to enhance and automate business processes has led to the development of Industry 4.0.	[110,111,112,113,114,115]
9	Environment monitoring	CPS implementation in geographical areas such as rivers, forests, etc., can be used for remote monitoring and quick response systems. The process of monitoring can be completed using minimal energy and no human intervention in the circumstances of natural and manmade disasters.	[116,117]
10	Security	The information collected from various sensors and other connected devices can be processed for fast decision making and enhancing security and privacy in a smart city ecosystem.	[118,119,120,121,122,123,124]
11	Smart homes	Smart homes are one of the most widely adopted applications of CPS. The various components of a regular household such as a security camera, electronic devices, home assistants, etc., are connected to automate the various processes.	[125,126,127,128,129,130]

**Table 2:** Security Attacks in CPS.

S.No	Ref	Focus Area	Threats	Mitigation
1	[134]	Context-Aware CPS security. Provided the notion of context-awareness in CPS.	False data injection, DoS attacks	Different categories of context concerning CPS were identified and a corresponding security mechanism was proposed
2	[135]	CPS in operational technology	Near realtime cyber attacks	Trap-based monitoring systems were proposed using a big data fusion model
3	[136]	CPS for smart grid	Malicious adversaries	STREAM approach for improving integrity and availability
4	[137]	CPS for healthcare	Data theft, impersonation, user profiling attacks, etc.	Cognitive cybersecurity framework using AI
5	[138]	WSN in CPS	Internal and external threats in WSN	Several mitigation approaches were discussed along with a comparison among them
6	[139]	Cross-domain CPS security	DoS and DDoS attacks, hacking, etc.	A security analysis framework was proposed identifying both discrete and continuous signal information flow in cross domain CPS
7	[140]	CPS for pervasive health monitoring systems	BAN attacks	PSKA and CAAC mechanisms were proposed
8	[141]	Securing big data in CPS	Identified various big data security issues	Discussed various security mechanisms for big data security using Weibull distribution.
9	[142]	Security of sensors in CPS	False data injection, spoofing, etc.	Discussed sensor attacks on edge and point of positions. Proposed classification of different kinds of attacks on sensors.
10	[143]	CPS for industrial processes	Deception attacks, DoS attacks, etc.	The concept of intrusion tolerance was proposed to secure industrial control systems.

## Data Availability

Not Applicable.
